# The Predominant Role of Musical Valence Over Arousal in Pain Modulation: A Psychophysiological Study

**DOI:** 10.1002/ijop.70142

**Published:** 2025-12-22

**Authors:** Veronika Diaz Abrahan, Guzmán Alba, Nadia Justel, Miguel A. Muñoz

**Affiliations:** ^1^ Laboratorio Interdisciplinario de Neurociencia Cognitiva (LINC), Centro de Investigación en Neurociencia y Neurospicología (CINN) Universidad de Palermo (UP), Consejo Nacional de Investigaciones Científicas y Técnicas (CONICET) Buenos Aires Argentina; ^2^ Mind, Brain and Behavior Research Center (CIMCYC) University of Granada Granada Spain; ^3^ Facultad de Psicología Universidad Autónoma de Madrid (UAM) Madrid Spain

**Keywords:** emotions, music listening, pain, pain modulation, valence‐by‐arousal interaction

## Abstract

Several studies have demonstrated the potential capacity of music to induce emotions and manage pain. However, the psychophysiological mechanisms underlying the effects of emotional dimensions (valence and arousal) induced by music on the modulation of pain perception remain poorly understood. In this research, we investigated the impact of the valence and arousal dimensions of music on the perception of pain intensity, aiming to discern which dimension has a greater influence. Healthy young participants were subjected to an acute heat pain stimulus, individually adjusted for each participant, whilst concurrently listening to musical excerpts categorised as pleasant, unpleasant or neutral. Pain ratings, the skin conductance response (SCR) and facial electromyographical (EMG) activity of the zygomaticus and corrugator muscles were recorded throughout the experimental task. After the experiment, subjective ratings in the valence and arousal dimensions were collected for each musical excerpt. In general, subjective measures and physiological correlates of emotions showed that selected musical excerpts elicited the expected affective responses. The pain intensity was greater when listening to unpleasant music than when listening to pleasant music. The main finding of the study indicates that the valence of music plays a more predominant role than arousal in pain modulation.

## 1 Introduction

Pain is defined as an unpleasant sensory and emotional phenomenon that involves sensory, affective, cognitive, social and biological components associated with actual or potential tissue damage (Feneberg et al. 2020; Merskey 1986). The main function of the pain processing system is to either prevent or address physical harm, activating defensive responses of withdrawal, avoidance or escape to dangerous stimuli (Peláez et al. 2019). Related research has focused on how affective stimuli may modulate the defensive pain response (Roy et al. 2012). According to the bioinformatics theory of emotion (Lang 1995), affective stimuli are organised around two basic affective dimensions: valence and arousal. The emotional valence dimension is related to liking and the hedonic value of stimuli, which extends from the unpleasant pole to the pleasant pole. The affective arousal dimension is related to the degree of emotional activation to realise the behaviour, which extends from the calm pole to the excited pole. When people are asked to judge the hedonic valence and arousal of a wide range of evocative stimuli, including pictures (Muñoz et al. 2013), sounds (Fernández‐Abascal et al. 2008), words (Sarli and Justel 2022) and music (Ortiz 2012; Tonini et al. 2024), the resulting distributions in the affective space are consistent with the motivational model. The distribution of stimuli along the valence and arousal dimensions formed a boomerang‐like pattern with two arms that extended from a neutral affective zone with minimum intensity to the two extremes of appetite and unpleasant maximum intensity (Bradley and Lang 1999). However, practical absence of stimuli in the low‐arousal, pleasant and unpleasant quadrants and in the high‐arousal and neutral quadrants were observed, indicating that affective stimuli that are very pleasant or very unpleasant elicit arousal to promote a behavioural response.

According to the motivational priming hypothesis (Lang 1995; Lang et al. 1990), pleasant stimuli activate the appetitive system, and unpleasant affective stimuli activate the aversive system. Thus, pleasant stimuli reduce defensive responses because activation of the appetitive system inhibits responses to aversive stimuli. In contrast, unpleasant stimuli increase defensive responses because of activation of the aversive system (Hackley et al. 2009; Muñoz et al. 2011). Similarly, pleasant pictures (Zidda et al. 2024) and films (Weisenberg et al. 1998) generally reduce the defensive pain response, whereas unpleasant stimuli increase it (Roy et al. 2012). Nevertheless, some studies have reported that unpleasant stimuli are effective at reducing pain reactions, whereas positive emotions lead to pain reduction as long as a minimal threshold of arousal is attained (Rhudy et al. 2004; Rhudy and Meagher 2001). These divergent findings may reflect the ongoing debate regarding which affective dimension – valence or arousal – exerts a greater influence on pain modulation (Rainville et al. 2005). For example, in a seminal study, Bradley et al. (2001) demonstrated that highly arousing stimuli, regardless of whether they are pleasant or unpleasant, enhance defensive reactions to a greater extent than low‐arousal stimuli do. This finding supports the view that emotional arousal, rather than valence, governs the modulation of defensive reflexes.

Several studies have shown the capacity of music to modulate pain perception (Roy et al. 2012, 2008), which affects the activity of brain areas related to the processing of pain (Martucci and MacKey 2018). Some researchers suggest that the analgesic effect of music could be associated with mood regulation more than with changes in arousal (Lu et al. 2019). A study conducted by Roy et al. (2012) evaluated the effects of music on pain ratings and skin conductance responses (SCRs) during electric shocks. The researchers compared the analgesic effects of three types of classical musical fragments (pleasant‐activating, pleasant‐relaxing and unpleasant‐activating) with those of a silent condition. The results indicated that there were no differences between pleasantly activating and pleasantly relaxing musical fragments, which suggests that the arousal level of music did not modulate pain. Surprisingly, although differences were found between unpleasant and pleasant musical fragments, they were not found between pleasant and silent conditions, indicating that the pleasantness of music did not have an analgesic effect. These results do not seem to confirm previous studies that demonstrated the analgesic effects of pleasant musical fragments when other pain stimuli were used (Roy et al. 2008). One explanation is that electric shocks induce negative affect related to fear responses and stress, masking the sensorial modulation of music. The literature suggests that responses obtained with electrical stimuli are more related to fear and negative expectations about electric shock than to pain itself (Nahman‐Averbuch et al. 2016). Electrical stimulation is unfamiliar and, because of personal concerns about possible adverse effects, is often accompanied by reports of anxiety and stress compared with thermal or mechanical stimulation (Lautenbacher and Rollman 1993; Rainville et al. 1992). Thus, as discussed by Sternbach (1968), although electric shock can effectively produce pain, it probably produces more significant alterations in the affective and evaluative dimensions, whereas thermal stimuli allow the experimental induction of pain with a relative absence of anxiety. Therefore, it is not surprising to find an analgesic effect of pleasant music when heat‐contact stimulation is used (Roy et al. 2008) but not when electrical stimulation is used (Roy et al. 2012).

The aim of the present study was to determine whether the valence or arousal dimension of stimuli exerts a greater influence on pain perception. We used a standard paradigm for the study of emotion (e.g., Bradley et al. 2001; Lang et al. 1993) to determine which of the two affective dimensions – valence and arousal – had greater weight on the modulation of defensive responses. In this paradigm, stimuli are selected to be high in arousal but differ in valence (pleasant stimuli with high‐arousal and unpleasant stimuli with high‐arousal), along with neutral stimuli characterised by low arousal. This approach allows for the dissociation of the relative contribution of each dimension to the modulation of pain. Thus, healthy participants listened to musical excerpts standardised in basic affective dimensions of valence and arousal. They could listen to pleasant (high‐valence, high‐arousal scores), unpleasant (low‐valence and high‐arousal scores) and neutral (medium valence and low‐arousal scores) musical excerpts. At the same time, acute thermal pain was administered, and after each trial, the participants rated the pain intensity. Facial electromyographical (EMG) activity (zygomaticus and corrugator) was recorded as a physiological measure of the hedonic value of the musical excerpts. Zygomaticus and corrugator activities allow us to distinguish between pleasant and unpleasant emotions (Larsen et al. 2003; Vrana 1993). In general, pleasant emotions activate the zygomaticus and inhibit corrugator activity (related to smiling), whereas unpleasant emotions activate the corrugator and inhibit zygomaticus activity (related to frowning). The SCR was recorded as a physiological measure of arousal, so higher levels of arousal are related to higher levels of SCR. Moreover, subjective ratings in the valence and arousal dimensions were collected for each music excerpt. Considering previous results (Roy et al. 2012, 2008), we expect that the valence dimension has a greater influence on pain modulation than does arousal. Therefore, pain responses are correlated with emotional valence ratings but not with emotional arousal ratings.

## 2 Materials and Methods

### 2.1 Participants

A total of 28 healthy volunteers (mean age = 22.42 years, SD = 0.28 years; 19 women) participated in the study. They were all students at the University of Granada, recruited via information provided in university classrooms, who received extra credit in return for their participation. Statistical estimations with G*Power (Faul et al. 2007) indicated that this sample size had sufficient statistical power (1 − *β* = 0.80) to detect differences between musical categories (pleasant, unpleasant and neutral) at a medium effect size (Cohen's *f* = 0.25, *α* error = 0.05 and assumed correlation of repeated measures = 0.5). None of the participants had psychiatric or neurological disorders or chronic pain or consumed analgesic medication or psychoactive substances before the study. The participant exclusion criteria also included visual or hearing impairment, amusia, music‐related pathology, chronic pain and cardiac pathologies. All the subjects signed informed consent forms to participate in the study, which was approved by the ethics committee of the University of Granada and was performed according to the recommendations of the Declaration of Helsinki.

### 2.2 Musical Excerpts

Twenty‐seven musical excerpts (nine pleasant‐activating, nine unpleasant‐activating and nine neutral‐non‐activating) were selected from two prior studies (Ortiz 2012; Vieillard et al. 2008). In both studies, many musical excerpts were evaluated via a 9‐point Likert scale on the valence and arousal dimensions of emotion. The procedures were similar across both studies: participants listened to musical excerpts ranging from 6 to 16.4 s in length and then rated each excerpt on the valence and arousal scales. The resulting distribution of musical excerpts across the two dimensions formed a characteristic ‘boomerang‐shaped’ pattern, with stimuli clustering along two arms extending from a neutral affective zone of minimal intensity towards high‐valence/high‐arousal (pleasant) and low‐valence/high‐arousal (unpleasant) zones (Bradley and Lang 1999; Fernández‐Abascal et al. 2008; Ortiz 2012; Tonini et al. 2024). Notably, both studies reported a practical absence of stimuli in the low‐arousal/unpleasant quadrant and in the high‐arousal/neutral quadrant, indicating a lack of highly pleasant or unpleasant stimuli that fail to elicit sufficient arousal to motivate a behavioural response. We selected pleasant, unpleasant and neutral musical excerpts with high, low and intermediate values of valence respectively (pleasant: 6.87 ± 0.52; unpleasant: 3.07 ± 0.54; neutral: 5.07 ± 0.45). With respect to arousal, pleasant and unpleasant musical excerpts were associated with high levels of arousal, whereas neutral musical excerpts were associated with low levels of arousal (pleasant: 7.43 ± 0.79; unpleasant: 7.32 ± 0.82; neutral: 2.76 ± 0.39).

Eighteen musical excerpts were used for the analysis of emotional modulation of pain (six pleasant excerpts: 58, g02, g03, g11, g13 and g14; six unpleasant excerpts: 3, p04, p07, p09, p11 and p14; and six neutral excerpts: 28, t01, t05, t11, t12 and t13). The remaining musical excerpts (9) were used to control predictability of pain stimulation and were presented without pain (three pleasant excerpts: 29, 43 and g10; three unpleasant excerpts: 2, 4 and p10; and three neutral excerpts: 38, 41 and t09). The musical excerpts had to be looped to achieve a duration of 14–19 s because the painful stimulus lasted for 12 s (as outlined in Section 2.5), and the length of the music excerpts ranged from 6 to 14 s. Thus, for example, music excerpts with a duration of 6 s were repeated for a total of 14 s, whereas 14 s musical excerpts were repeated for a total of 19 s.

### 2.3 Pain Stimuli

We used thermal stimulation produced by a 4 × 4 cm Peltier plate as painful stimuli. Thermal stimulation is a safe, reliable and frequently applied method to induce acute pain. The participants' pain tolerance was assessed using the method of limits (Alba et al. 2022). The sequence of pain assessments was as follows. First, the participants were instructed to keep the index finger of their left hand in contact with the thermode at 37°C for 5 s. Then, the participants had to rate the unpleasantness of the temperature using a 0–10 visual analogue scale (VAS; 0 represented ‘no unpleasant temperature’, 5 represented ‘the temperature is starting to become unpleasant’ and 10 represented ‘the unpleasantness of the temperature is unbearable’). After the prior temperature was rated, the next temperature (1°C warmer than the previous temperature) was delivered and evaluated. When the participants rated the unpleasantness of the heat stimulus at 10, the procedure was stopped, and the temperature value was recorded. This procedure was repeated three times for each participant, and the average of the three temperature values was recorded as the individual's heat pain tolerance. The heat pain stimulus for each participant was calculated as 60% of that individual's heat pain tolerance (i.e., heat pain stimulus temperature = [0.6 × {average heat pain tolerance − 37°C} + 37°C]).

### 2.4 VAS and Self‐Assessment Manikin (SAM)

Pain intensity was evaluated via a 10‐point computerised version of the VAS (0 = no pain; 10 = unbearable pain). The valence and arousal of each musical excerpt were evaluated via a computerised version of the SAM (Muñoz et al. 2013). The SAM is a non‐verbal pictographic assessment technique that measures the subjective valence (unpleasant–pleasant) and arousal (excited–calm) associated with a person's affective reaction to a wide variety of stimuli. Each SAM scale is represented by five humanoid figures (and four intervals between figures) ranging from a frowning face to a smiling face (for the valence dimension) and from a sleepy‐looking face to an agitated figure (for the arousal dimension). The ratings are converted to numbers ranging from 1 (very unpleasant/very calm) to 9 (very pleasant/very excited). The valence dimension is defined as a bipolar scale with one extreme as pleasant (‘if you feel happy, pleased, satisfied, contented, hopeful while listening to the music, you should choose a rating of 9’) and the other as unpleasant (‘if you feel completely unhappy, annoyed, unsatisfied, melancholic, despaired, bored, you should choose a rating of 1’). The arousal dimension is defined as a monopolar scale with one extreme as excited (‘if you feel stimulated, excited, frenzied, jittery, wide‐awake, aroused while listening to the music, you should choose the rating 9’) and the other as calm (‘If you feel completely relaxed, calm, sluggish, dull, sleepy, unaroused, you should choose the rating 1’). The participants were asked to respond how they felt whilst listening to the music excerpt.

### 2.5 Procedure

**FIGURE 1:**
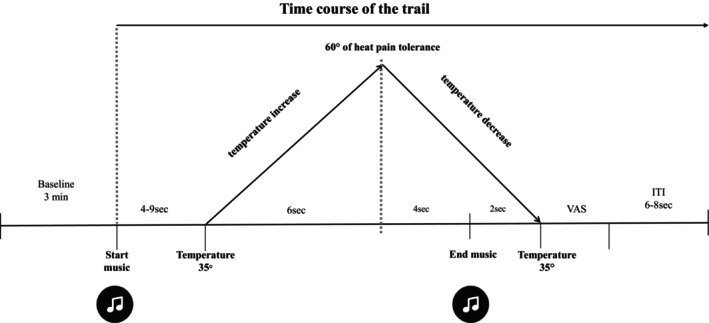
Experimental procedure.

Data were compiled in an individual session that lasted approximately 50 min (see Figure 1). Upon their arrival at the laboratory, the participants received a brief description of the study prior to providing their written informed consent. Then, they completed a short interview to verify their compliance with the inclusion criteria and to assess their sociodemographic features. Next, the participants were moved to an electrically shielded, quiet and dimly illuminated room, where they settled comfortably in an armchair in front of an LED screen.

Prior to starting the experiment, participants' pain tolerance was assessed using the method of limits to assess heat pain tolerance (see above). The participants had a pain tolerance lower than the 60°C painful stimulus (mean: 53.54 ± 5.24). Subsequently, EMG and SCR electrodes, as well as headphones, were placed. The participants were instructed to keep the major finger of their left hand in contact with the thermal plate until pain became unbearable, to pay attention to the music that they were listening to and to evaluate pain intensity at the end of each trial. After the instructions, the experimental session started.

The experimental session consisted of an adaptation period of 3 min followed by 48 experimental trials. The task included 36 trials of music with pain stimulation: the 18 musical excerpts + pain (6 pleasant, 6 unpleasant and 6 neutral) presented two times each. Moreover, we included 12 trials to avoid predictability of pain stimulation: 9 trials of music without pain (3 pleasant, 3 unpleasant and 3 neutral) and 3 trials of silence with pain. The trial presentation was counterbalanced across three orders; thus, all the participants heard the same musical excerpts, albeit in a distinct order. No affective category was repeated consecutively; for example, a trial with a pleasant music excerpt was followed by a neutral or unpleasant music excerpt. Silence trials were presented every 12 trials, and music without pain trials were presented randomly every 4–6 trials. The sequences were strategically structured: order 1 started with a pleasant trial, order 2 started with an unpleasant trial and order 3 started with a neutral trial. At the end of each trial, the participant had unlimited time to rate the level of pain intensity via the VAS (0 = not pain; 10 = unbearable pain).

Each music + pain trial began with the presentation of 4–9 s of musical stimulation followed by 12 s of pain stimulation. The pain stimulus started to increase from 35°C to 60% of the individual's heat pain tolerance for 6 s, after which the temperature decreased to 35°C again. Muscular fragments ended 4 s after the maximum peak temperature was reached. The participants were informed that they could remove their finger from the plate if pain became unbearable and that they could put their finger back on the plate after rating the pain intensity. The intertrial interval (ITI) between trials oscillated between 6 and 8 s.

After the completion of the task, the electrodes were retired, and the participants listened to the 36 musical excerpts, which were presented with pain once again, and evaluated each excerpt in the valence and arousal dimensions with a computerised version of the SAM.

### 2.6 Physiological Measurements

Physiological measurements were recorded via a BIOPAC amplifier (MP150; BIOPAC Systems Inc., Goleta, CA, USA) set at a sample rate of 1000 samples per second and a notch filter at 50 Hz. The acquisition was controlled by BIOPAC's AcqKnowledge software Version 3.0.21 (Mindware Technologies Ltd., Gahanna, OH, USA).

#### 2.6.1 Affective Response to Musical Excerpts

To examine the affective response to music, facial EMGs and SCRs were analysed during the first 4 s following music onset in the music + pain trials. This time window was selected to equalise the analysis of physiological responses, although pain stimulation can occur at any point between 4 and 9 s after music onset.

EMG activity was recorded with bipolar Ag‐AgCl electrodes attached over the zygomaticus and corrugator muscles and grounded to the left elbow. Direct EMGs were recorded via a bandpass filter of 100–500 Hz. The data were analysed offline via a MATLAB script that rectified and integrated the signal (in μV) with a time constant of 500 ms over an interval starting 100 ms before the trial and lasting for 4 s following music onset. Facial EMG activity in the zygomaticus and corrugator magnitude was defined as the mean amplitude of the integrated EMG response for 4 s. Those trials that were not higher than the mean of baseline were computed as zero microvolts (V). One participant was excluded from the corrugator muscle analyses due to artefacts in the recording (electrode pop‐up, cable movement or participant movements).

For the SCR recordings, two Ag‐AgCl electrodes were placed in the hypothenar eminence of the left hand and measured in μS. The data were analysed offline via a MATLAB script, which averaged the signals for the first 4 s of music + pain trial onset. To eliminate basal levels of SCR, the data were transformed into differential scores by subtracting the average SCR during the 3 s before the music + pain trial started. Five participants were excluded because recording issues resulted in a flat SCR response, making it impossible to conduct a proper analysis.

### 2.7 Statistical Analyses

All analyses were performed over the 36 music + pain trials, which were formed by 18 musical fragments (6 pleasant, 6 unpleasant and 6 neutral) presented two times. Two groups of analyses were performed via repeated measures analysis of variance (ANOVA). The first group was about affective response to musical fragments, including the analysis of SAM, facial EMG and SCR. Ratings of SAM valence and arousal were separately analysed via repeated measures ANOVAs with CATEGORY (pleasant, unpleasant and neutral musical excerpts) as the single within‐subject factor. EMG activity of the zygomatic and corrugator muscles was analysed by an ANOVA 3 × 3 with ORDER (three counterbalanced orders) as a between‐group factor and CATEGORY (pleasant, unpleasant and neutral) as a within‐subject factor. The analysis of the SCR was equal to the analysis of the EMG response via 3 × 3 repeated measure ANOVA (ORDER by CATEGORY).

The second group of analyses focused on how different musical categories modulated pain perception, potentially through emotional mechanisms. To this end, we analysed pain intensity ratings (VASs) via 3 × 2 ANOVA, with CATEGORY (pleasant, unpleasant and neutral) and pain intensity ratings (TIME) (first and second presentations of the same music excerpt) as within‐subject factors. Finally, Pearson correlations were calculated between the SAM valence and arousal scores and the means of the two evaluations of the VAS scores.

In all analyses, the Greenhouse–Geisser epsilon correction was applied to control for violation of the sphericity assumption. The results are reported with the original degrees of freedom and the corrected *p* values. When significant effects were found, post hoc analyses were performed via Bonferroni correction, with the level of significance set at 0.5. Partial eta squared ($\eta_{p}^{2}$) was used as the effect size for the *F* tests. All analyses were performed with SPSS v.23.0 (SPSS Inc., Chicago, IL, USA).

## 3 Results

### 3.1 Affective Response to Music

**FIGURE 2:**
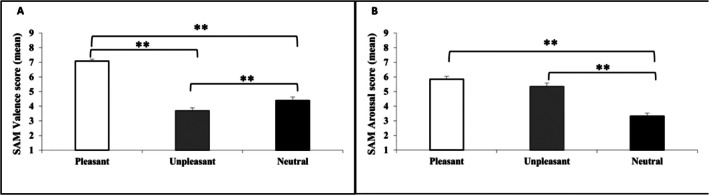
Affective response to music fragments. (A) Means of SAM valence scores for pleasant, unpleasant and neutral musical excerpts. (B) Means of SAM arousal scores for pleasant, unpleasant and neutral musical excerpts. **p < 0.01.

Table 1 shows the ratings of SAM valence and arousal in each musical excerpt. For each of the SAM measures (valence and arousal), we conducted two repeated measures ANOVAs with CATEGORY (pleasant, unpleasant and neutral musical excerpts) as the single within‐subject factor. The analysis confirmed that the chosen excerpts induced the expected emotions. With respect to valence, the results confirmed a significant CATEGORY effect, *F*(2, 54) = 130.81, *p* < 0.01, $\eta_{p}^{2}=0.83$. The pairwise test with Bonferroni correction revealed that the valence score was higher for pleasant musical excerpts than for neutral and unpleasant musical excerpts. The valence score in the neutral category was higher than the score in the unpleasant musical excerpts category (all *p* < 0.01; Figure 2A). In relation to the arousal dimension, repeated measures ANOVAs yielded significant differences in CATEGORY, *F*(2, 54) = 49.60, *p* < 0.01, $\eta_{p}^{2}=0.65$. The pairwise comparisons revealed that the arousal score was higher for pleasant and unpleasant musical excerpts than for neutral ones (all *p* < 0.001). No significant differences were found between pleasant and unpleasant excerpts in the arousal dimension (Figure 2B).

**TABLE 1:** Valence, arousal and pain scores for each musical excerpt.

				Pain intensity		
		Valence	Arousal	First presentation	Second presentation	Total
Musical excerpt	Category	Mean (SD)	Mean (SD)	Mean (SD)	Mean (SD)	Mean (SD)
29^b^	Pleasant	7.11 (1.31)	6.75 (1.35)			
g14^a^	Pleasant	6.96 (1.50)	4.86 (2.24)	2.86 (2.40)	3.07 (2.19)	2.96 (2)
g11^a^	Pleasant	7.07 (1.15)	4.64 (1.59)	2.93 (2.28)	2.79 (2.39)	2.86 (2.08)
g03^a^	Pleasant	7.04 (1.37)	5.00 (2.11)	3.93 (3.05)	2.71 (2.29)	3.32 (2.44)
g13^a^	Pleasant	7.29 (1.12)	5.54 (1.75)	3.32 (2.44)	3.21 (2.03)	3.27 (1.97)
g10^a^	Pleasant	7.43 (1.03)	6.50 (1.73)			
58^b^	Pleasant	6.00 (1.76)	5.93 (1.86)	3.71 (2.07)	2.57 (1.88)	3.14 (1.58)
43^b^	Pleasant	7.25 (1.62)	7.86 (1.27)			
g02^a^	Pleasant	7.61 (1.10)	5.54 (1.67)	2.86 (2.48)	3.21 (2.28)	3.04 (2.06)
Total mean (SD)		**7.08 (0.68)**	**5.85 (1.08)**			**3.10 (1.74)**
4^b^	Unpleasant	3.75 (1.76)	6.71 (1.49)			
2^b^	Unpleasant	2.82 (1.59)	7.04 (2.01)			
p09^a^	Unpleasant	3.46 (1.64)	3.68 (2.09)	3.46 (2.29)	3.71 (2.28)	3.59 (1.92)
p11^a^	Unpleasant	3.64 (1.47)	4.43 (1.83)	3.29 (2.21)	3.64 (2.38)	3.46 (2.07)
p10^a^	Unpleasant	3.50 (1.32)	4.39 (1.93)			
p04^a^	Unpleasant	4.43 (1.14)	5.11 (1.79)	3.64 (2.15)	3.50 (2.47)	3.57 (2.10)
3^b^	Unpleasant	2.93 (1.61)	7.11 (1.87)	3.96 (2.67)	4.29 (2.19)	4.13 (2.13)
p07^a^	Unpleasant	4.71 (1.27)	4.79 (1.91)	2.68 (2.11)	3.50 (2.30)	3.09 (1.74)
p12^a^	Unpleasant	3.93 (1.44)	4.79 (1.81)	4.25 (1.86)	3.00 (2.28)	3.62 (1.84)
Total mean (SD)		**3.69 (0.92)**	**5.34 (1.32)**			**3.58 (1.60)**
28^b^	Neutral	6.07 (1.90)	2.79 (1.77)	3.32 (2.51)	2.86 (2.17)	3.09 (1.87)
t13^a^	Neutral	3.07 (1.82)	3.25 (1.82)	3.39 (2.18)	3.50 (2.17)	3.45 (1.74)
38^b^	Neutral	5.04 (1.86)	3.54 (2.12)			
t12^a^	Neutral	4.11 (1.99)	3.36 (1.87)	3.14 (1.80)	3.04 (1.90)	3.09 (1.85)
t11^a^	Neutral	3.14 (1.33)	3.11 (1.59)	3.18 (2.13)	3.50 (2.43)	3.34 (2.07)
t09^a^	Neutral	4.07 (1.76)	3.86 (1.94)			
t01^a^	Neutral	3.29 (1.88)	3.11 (1.52)	3.21 (2.18)	3.61 (2.10)	3.41 (1.88)
41^b^	Neutral	6.50 (2.20)	3.71 (2.19)			
t05^a^	Neutral	4.25 (1.53)	3.11 (1.62)	4.21 (2.35)	2.43 (1.99)	3.32 (1.62)
Total mean (SD)		**4.39 (1.08)**	**3.31 (1.15)**			**3.28 (1.51)**

*Note:* Means and standard deviations of the valence, arousal and pain intensity perceived (VAS) scores. Blank space corresponds to fragments without pain.

^a^ Vieillard et al. (2008).

^b^ Ortiz (2012).

**FIGURE 3:**
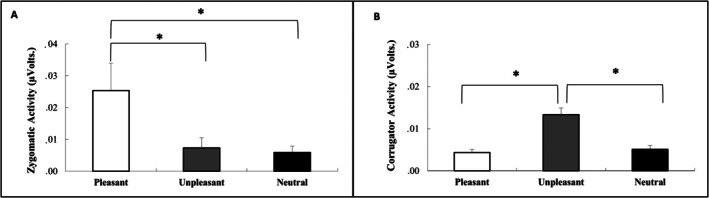
Facial electromyographical activity. (A) Means of EMG zygomatic activity (volts scores) for pleasant, unpleasant and neutral musical excerpts. (B) Means of EMG corrugator activity (volts scores) for pleasant, unpleasant and neutral musical excerpts. *p < 0.01.

For EMG zygomatic activity for 4 s before pain stimulation, 3 × 3 repeated measures ANOVA (ORDER by CATEGORY) yielded a significant main effect for CATEGORY, *F*(2, 42) = 7.86, *p* < 0.01, $\eta_{p}^{2}=0.27$. The factors ORDER and the interaction between ORDER and CATEGORY were not significant (*p* > 0.05). Pairwise comparisons with Bonferroni correction comparing the three affective categories (pleasant, unpleasant and neutral) revealed that zygomaticus activity was greater in pleasant music trials than in neutral and unpleasant trials (*p* < 0.05). No significant differences were found between neutral and unpleasant musical trials. In line with the valence scores, pleasant music evoked greater facial expressions of pleasantness than did unpleasant and neutral music (Figure 3A).

In relation to EMG corrugator activity, the 3 × 3 repeated measure ANOVA (ORDER by CATEGORY) yielded a significant difference for CATEGORY, *F*(2, 40) = 41.39, *p* < 0.01, $\eta_{p}^{2}=0.67$. The main factor of ORDER and the interaction between ORDER and CATEGORY were not significant (all *p* > 0.05). Pairwise comparisons with Bonferroni correction comparing the three affective categories (pleasant, unpleasant and neutral) revealed that corrugator activity was greater in unpleasant music trials than in neutral and pleasant trials (all *p* < 0.01). No significant differences were found between neutral and pleasant musical fragments. These results confirmed that unpleasant music evoked greater unpleasantness than pleasant and neutral music did.

The ANOVA 3 × 3 (ORDER by CATEGORY) conducted on the SCR response revealed a marginal effect of CATEGORY, *F*(2, 32) = 2.94, *p* = 0.06, $\eta_{p}^{2}=0.16$ (pleasant *M* = −0.003, SD = 0.08; unpleasant *M* = 0.015, SD = 0.12; neutral *M* = −0.016, SD = 0.10). The main factor, ORDER, and the interaction between ORDER and CATEGORY were not significant (all *p* > 0.05).

### 3.2 Emotional Modulation of Pain

**FIGURE 4:**
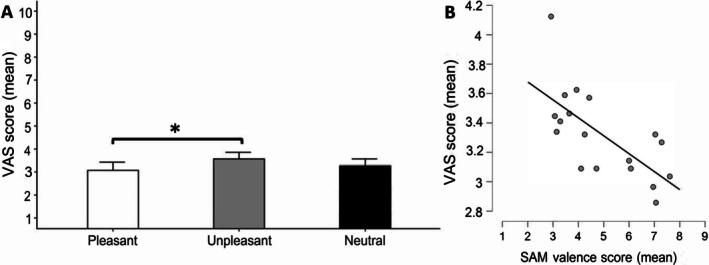
Pain perception. (A) VAS score results. (B) VAS scores and SAM correlations. Means of VAS scores in pleasant, unpleasant and neutral musical excerpts during pain stimulation. *p < 0.01.

The results of ANOVA 3 × 2 (CATEGORY by TIME) over the scores of perceived pain intensity (VAS) revealed a significant main effect of CATEGORY, *F*(2, 54) = 6.93, *p* < 0.01, $\eta_{p}^{2}=0.20$. The corresponding pairwise comparisons indicated that participants perceived pain as less intense during listening to pleasant musical trials than after listening to unpleasant trials (*p* < 0.05; see Figure 4A). No significant differences in VAS scores were found between neutral and other affective musical trials (*p* < 0.05; see Figure 4A). The main factor of the TIME factor and the interaction CATEGORY by TIME were not significant (*p* > 0.05).

Pearson correlations between VAS and SAM valence and arousal scores yielded a significant negative correlation between VAS scores and SAM valence (*r* = −0.68; *p* < 0.01). Thus, as the valence level increased, the pain intensity score decreased (Figure 4B). No significant correlation was found between the VAS score and arousal scale score (*r* = 0.26; *p* > 0.05).

## 4 Discussion

The aim of the present study was to determine whether the valence or arousal dimension of musical excerpts has a greater influence on pain modulation. Healthy participants listened to pleasant, unpleasant and neutral musical excerpts whilst thermal pain was present. To maximise the differential effects of valence and arousal, pleasant and unpleasant excerpts were matched for arousal, differing only in valence, whereas neutral stimuli differed in valence and maximally in arousal from the other two musical categories. Moreover, musical excerpts were scored on SAM valence and arousal scales, and self‐reported pain ratings and EMG facial and SCR activities were recorded whilst healthy participants listened to musical excerpts and a pain stimulus was applied.

In relation to the affective response to standardised musical excerpts, as expected, SAM valence ratings revealed that pleasant music evoked greater pleasantness than did unpleasant and neutral music, whereas unpleasant music evoked greater unpleasantness than did pleasant and neutral music amongst our participants. These results were confirmed by EMG measurements: pleasant music evoked greater zygomatic (muscle associated with smiling) activity than did unpleasant and neutral music. However, corrugator (muscle associated with frowning) activity was greater for unpleasant musical excerpts than for pleasant and neutral musical excerpts. Our results replicate previous findings on listening to affective music (Fuentes‐Sánchez et al. 2021; Roy et al. 2009), providing additional empirical support for the underlying activation of the appetitive system during pleasant music and the activation of the aversive system during unpleasant music. As expected, the SAM arousal ratings revealed that the pleasant and unpleasant music excerpts were more arousing than the neutral excerpts were, without significant differences between the pleasant and unpleasant excerpts. The SCR results revealed a marginal effect between emotional categories, indicating that neutral music excerpts produced lower physiological arousal than pleasant and unpleasant excerpts did. In general, an increase in the SCR is associated with increased physiological arousal, which is usually caused by emotional stimuli (Lang et al. 1993), both pleasantly and unpleasantly. The lack of an SCR could indicate that the SCR may require more musical exposure to become evoked. SCR is a slow physiological response and typically takes several seconds to observe changes associated with emotional responses (Dawson et al. 2017). Increasing the exposure time of each stimulus could clarify the possible differences in affective response between neutral and emotional music.

The principal aim of this work was to determine which basic affective dimensions (valence or arousal) exert greater influence on pain perception. The pain ratings revealed that the participants perceived the painful stimulus as more intensive when they listened to unpleasant music than when they listened to pleasant musical excerpts. Unpleasant and pleasant music were matched in terms of arousal. Thus, the differences between the effects of unpleasant and pleasant music on pain modulation are explained by the valence dimension and not by arousal. These results were reinforced by the negative correlation between the VAS score and valence score coupled with the lack of correlation between the VAS score and arousal score. Our findings are in line with Roy's group findings, where a decrease in valence was associated with an increase in the intensity and unpleasantness of painful stimuli (Roy et al. 2012, 2008). Unpleasant music probably increases pain intensity because both activate the aversive system, thereby increasing the defensive response (Lang 1995). This finding is in line with articles that reported that pleasant music has an analgesic effect on pain perception (Roy et al. 2008). We partially confirm this evidence. When contrasting unpleasant and pleasant music, participants reported reduced pain intensity perceptions after listening to pleasant music. Thus, responses of the defensive system could be inhibited by pleasant stimuli, resulting in a reduction in pain perception. However, the lack of differences between pleasant and neutral musical excerpts raises questions about the analgesic effect of pleasant music. The results of the SAM by musical excerpt showed that four neutral musical excerpts were not perceived as neutral by the participants (28, t01, t11 and t13). We try to include neutral music fragments under parameters used in studies with emotional images (Bradley and Lang 1999), but it is possible that the parameters for the selection of emotional music pieces can differ. Some authors have even suggested that music is a complex phenomenon and that neutral music does not exist (Peretz et al. 1998). All music generates an emotional response. However, recent research suggests that individual music preferences could play a significant role in the analgesic effect of music (Basiński et al. 2021; Howlin and Rooney 2021); for example, Van der Valk Bouman et al. (2024) reported that pain tolerance increases significantly when participants listen to music from their preferred genre. In contrast, Merrill et al. (2023) reported that disliked music elicits heightened physiological responses, including increased heart rate and facial expressions of distress, suggesting that non‐preferred music may act as a stressor. Similarly, Howlin and Rooney (2021b) demonstrated that increased perceived control over music selection is associated with greater pain tolerance, and Howlin et al. (2022) reported that active engagement with music predicted reductions in pain intensity. These results underscore that music is not a universally effective stimulus: its analgesic and emotional effects are highly dependent on individual preferences and perceived control. Future research should therefore incorporate measures of music preference and allow participants to select music that is personally meaningful to maximise the therapeutic potential of music interventions. Furthermore, we preselected classical music, which could be less exciting than other musical genres. These findings could explain why the analgesic effect of pleasant music was small in the present study (Basiński et al. 2021).

One important limitation of the present study is the absence of pleasant and unpleasant low‐arousal musical excerpts, such as those used by Roy et al. (2008). Importantly, the distribution of musical excerpts in our set followed a boomerang‐shaped pattern, which is consistent with the affective norms in both music and emotional image databases (Bradley et al. 2001); this naturally results in gaps in the affective space, particularly in the pleasant low‐arousal and unpleasant low‐arousal quadrants. Moreover, when the goal is to emphasise the distinct contribution of valence and arousal dimensions, it is common practice to use extreme stimuli (i.e., highly pleasant or unpleasant with strong arousal contrasts), as these maximise emotional reactivity and allow for clearer differentiation of effects (Roy et al. 2008). Whilst our design emphasised this strategy, we acknowledge that the inclusion of pleasant low‐arousal music would have enriched the design and provided a more complete representation of the affective space.

## 5 General Conclusion

It is well known that listening to music can reduce pain perception and regulate affective responses (Feneberg et al. 2020). Our results show that pain intensity is greater when listening to unpleasant music than when listening to pleasant music. In conclusion, these findings support the hypothesis that the emotional valence of music has a greater influence than the arousal dimension on pain modulation (Roy et al. 2008). Music can induce emotions that can reduce pain perception.

## Author Contributions

**Veronika Diaz Abrahan:** investigation (lead) – performing the experiments, formal analysis, design of methodology, and writing – original draft preparation (equal). **Guzmán Alba:** software, design of methodology, and writing – original draft preparation (equal). **Nadia Justel:** supervision, writing – original draft preparation (equal), and funding acquisition. **Miguel A. Muñoz:** supervision, writing – original draft preparation, conceptualization (lead), design of methodology, provision of study materials, writing – review and editing, and funding acquisition.

## Funding

This work was supported by Ministerio de Economía y Competitividad [Spanish Ministry of Economy and Competitiveness] (Grant No. PSI2017‐88388‐C4‐3‐R), Consejería de Economía, Conocimiento, Empresas y Universidad, Junta de Andalucía [Andalusian Regional Ministry of Economic Transformation, Industry, Knowledge, and Universities] (Grant No. B‐SEJ‐028‐UGR18) and Universidad Nacional de San Martín (Grant Nos. PICT 2014‐1323.2015‐2017, PICT 2017‐0558.2018‐2021).

## Ethics Statement

All procedures performed in studies involving human participants were in accordance with the ethical standards of the Granada University Research Ethics Committee and with the 1964 Helsinki Declaration and its later amendments or comparable ethical standards.

## Consent

As noted in the procedure of the current study, informed consent was obtained from all participants included in this study.

## Conflicts of Interest

The authors declare no conflicts of interest.

## Data Availability

The data that support the findings of this study are available on request from the corresponding author. The data are not publicly available due to privacy or ethical restrictions.
